# Healthcare Service Utilization and Medication Use in 128,239 Children with Atopic Dermatitis in Israel—A Cross-Sectional Case-Control Study

**DOI:** 10.3390/jcm14186402

**Published:** 2025-09-10

**Authors:** Naama Tova Cohen, Amit Iton-Schwartz, Doron Comaneshter, Yulia Valdman-Grinshpoun

**Affiliations:** 1 Azrieli Faculty of Medicine, Bar-Ilan University, Safed 1311502, Israel; 2 Division of Dermatology, Rabin Medical Center, Petah Tikva 4941492, Israel; 3 Clalit Health Services, Tel Aviv 6209804, Israel; 4 Department of Dermatology, Soroka Medical Center, Be’er Sheva 8410101, Israel; 5 Faculty of Health Sciences, Ben Gurion University of the Negev, Be’er Sheva 8410501, Israel

**Keywords:** atopic dermatitis, pediatric, biologic agents, epidemiology

## Abstract

**Background:** Atopic dermatitis (AD) is a chronic inflammatory disease requiring topical and systemic treatments. This study examines healthcare service utilization and medication use in children with AD in a large healthcare organization during the year 2024. **Methods:** A cross-sectional case-control study was conducted comparing 128,239 children with AD to 128,239 matched controls regarding healthcare utilization and medication use. Multivariate analysis assessed differences between the groups. **Results:** Children with AD had increased healthcare utilization compared to the control group, with higher rates of visits to pediatricians, general practitioners, and dermatologists. A total of 144 children (0.11%) with AD were treated by immunosuppressive drugs, as compared to 78 children (0.06%) in the control group (OR 1.8, 95% CI 1.4–2.4, *p*-value < 0.001). A total of 410 children (0.32%) were treated with biologic drugs as compared to 12 children (0.01%) in the control group (OR 34.3, 95% CI 19.3–60.9, *p*-value < 0.001). A total of 34 children (0.03%) were treated with Janus kinase (JAK) inhibitors as compared to 2 children (0.002%) in the control group (OR 17, 95% CI 4.1–70.8, *p*-value < 0.001). **Conclusions:** Increased utilization of healthcare services was observed in pediatric patients with AD compared to the control group. As only a small proportion of the children with AD received immunosuppressants, biologic treatments, and JAK inhibitors, we suggest that the use of systemic medications should be strongly considered in pediatric patients with moderate to severe AD.

## 1. Introduction

Atopic dermatitis (AD) is a chronic inflammatory disorder that begins in early childhood, with the majority of patients presenting before the age of five years [1]. In most cases, symptoms persist beyond infancy and continue into later childhood or adolescence [2,3]. The pathogenesis of AD is multifactorial, involving genetic susceptibility, epidermal barrier dysfunction, and immune dysregulation—all of which contribute to disease development and chronicity [4].

Management of AD is directed by disease severity. First-line therapy includes emollients and topical anti-inflammatory agents, such as topical corticosteroids (TCS), topical calcineurin inhibitors (TCI), and phosphodiesterase-4 inhibitors (PDE4). For moderate-to-severe disease, the treatment includes conventional systemic immunosuppressive agents, small molecules agents, and biologic agents [5]. In primary care settings, pediatric patients with AD present a significant clinical and therapeutic burden requiring a multifaceted treatment approach [6].

Recent advances in understanding AD pathophysiology have enabled the development of targeted therapies, including biologic agents and JAK inhibitors, representing a major milestone in the management of moderate-to-severe AD in both adult and pediatric patients [7,8]. These agents have shown efficacy and favorable safety profiles in pediatric clinical trials; however, data about their real-world use in pediatric settings remain limited [9,10]. Understanding real-world treatment patterns is necessary to optimize disease management in clinical practice.

This study aims to assess healthcare service utilization and medication use patterns among children with AD in Israel. Specifically, we aim to explore the use of systemic medications in pediatric patients with AD.

## 2. Materials and Methods

### 2.1. Setting and Data Sources

Clalit Health Services (CHS) is the largest Israeli healthcare provider for over 50% of the Israeli population (about 5 million people in June 2025). CHS provides healthcare services across all geographic regions of Israel, including urban, suburban, and rural areas, and serves a diverse population in terms of ethnicity, socioeconomic status, and cultural background (Table S1, Supplementary Materials). Dermatology consultations at CHS are provided at inpatient hospital settings, in outpatient clinics affiliated with hospitals, or at community-based clinics (about 1.3 million dermatology visits per year). The community clinics are operated by board-certified dermatologists and are classified as primary care services—there is no need for a referral letter for Clalit enrollees for community dermatology visit.

At CHS, all prescribed medications, including TCS, TCI, PDE4, and systemic and biologic treatments, are documented through electronic prescriptions, and subsequent purchases are recorded in the claiming system.

### 2.2. Study Population and Matching Process

A cross-sectional study was conducted comparing AD pediatric patients to a control group without AD, matched by age, sex, and primary clinic address, during the year 2024. A thorough review of the CHS dataset was conducted to identify all pediatric patients diagnosed with AD during 2024. Patients with a documented AD diagnosis recorded by a dermatologist were included in the study. Data were extracted from the centralized health record system of CHS, which includes all medical encounters, visits in both hospital and community setting laboratory tests, and imaging results. Data were linked via the Israeli national identification number, enabling a comprehensive retrieval of clinical and healthcare utilization data.

### 2.3. Outcome Assessment

Demographic and clinical information extracted from the CHS medical database included age, sex, smoking status, ethnicity (Arab and non-Arab), body mass index (BMI), and severity of AD as defined according to healthcare utilization, as described in the Section 2.4.

Healthcare service utilization data included community clinic visits (general practitioner and pediatric clinic visits) and professional community consultations (internal medicine, allergy, dermatology, ophthalmology, otolaryngology, and pulmonology clinic visits) during the year 2024. In- and out-patient service consumption included emergency room (ER) visits and the total number of hospital visits and hospitalization during the year 2024.

Drug use data and pharmacy claims included both topical and systemic treatments. Topical treatments included TCS, TCI, and a PDE4 inhibitor. Systemic treatments included antihistamines, systemic corticosteroids, immunosuppressive drugs (methotrexate, ciclosporin, azathioprine, and mycophenolate mofetil), biologic drugs (dupilumab), and a JAK inhibitor (upadacitinib), since this was the only JAK inhibitor available for patients under the age of 18 years during 2024 in Israel. Phototherapy included narrow-band UVB. The extraction of drug utilization data was based on the Anatomical Therapeutic Chemical Classification.

### 2.4. Severity

Disease severity scoring systems for AD, such as the Severity Scoring of Atopic Dermatitis (SCORAD) or investigator’s global assessment (IGA), are widely used in clinical trial settings. However, in routine clinical practice in CHS, AD severity is typically assessed based on symptoms and the extent of eczema, and scoring is absent. Therefore, as conducted in previous study [11], dispensed AD-related pharmacologic treatments were utilized as a surrogate measure for disease severity in our study, as follows:

Mild AD: A maximum of one month of topical corticosteroid use.

Moderate AD: At least one of the following:

- At least two months of topical corticosteroid use.
- At least one month of topical calcineurin inhibitor/topical PDE4 inhibitor.
- At least one course of phototherapy treatment.

Severe AD: Any use of a systemic agent (systemic immunosuppressive agents, biologic, JAK inhibitors).

As systemic corticosteroids are utilized for a broad spectrum of indications, including acute and self-limited conditions, their use was excluded from the severity classification to avoid potential misclassification bias.

### 2.5. Statistical Analysis

Healthcare utilization and drug use were calculated per patient in the case and control group, and were compared between cases and controls in univariate analyses and multivariate analyses, adjusting to confounding parameters. The study population was subdivided according to age. Data were described for the entire study population and for each subgroup.

Continuous variables were compared using *t*-tests for independent samples, and dichotomous variables were compared by Pearson’s chi-squared test, as applicable.

The association between AD patients and the burden of their use of healthcare and drug services was tested by a univariate analysis and a multivariate logistic regression model which included several explanatory parameters. Statistical analysis was performed using IBM SPSS statistics for Windows software, version 29.02 (Armonk, NY, USA), and a *p*-value less than 0.05 was considered statistically significant.

## 3. Results

### 3.1. Characteristics of the Study Population

The study included 128,239 pediatric patients (age 0–18 years) with AD and 128,239 age-, sex-, primary clinic-matched controls. Clinical characteristics of the study population are presented in Table 1. The mean age was 9.7 years (SD 4.9), and 47.4% were females. The age distribution of the patients was as follows: 18.0% at age 0–4 years, 30.4% at age 5–9 years, 30.1% at age 10–14 years, and 21.6% at age 14–18 years. According to our method of severity assessment, most patients (89.4%) had mild AD, 10% had moderate AD, and only 0.42% presented with severe disease (Table 1). In total, 5.7% of children with AD had asthma, as compared with 3.3% in the control group (*p*-value < 0.001).

### 3.2. Healthcare Service Utilization

Healthcare service utilization among children with AD and controls during 2024 is detailed in Table 2, which includes both the mean number of visits per patient and the odds ratios (ORs) for healthcare service utilization. Children with AD had an increased number of healthcare visits per year (2024) as compared to the control group, including general practitioner (1.8 vs. 1.5), pediatric clinic visits (4.8 vs. 3.8), and visits to specialist community clinics, such as dermatology (0.5 vs. 0.2), allergy (0.05 vs. 0.02), pulmonology (0.02 vs. 0.0), and ophthalmology (0.23 vs. 0.18). Furthermore, children with AD had increased odds of utilizing healthcare services compared to the control group, including dermatology clinics (OR 2.8, 95% CI 2.8–2.9), allergologists (OR 2.8, 95% CI 2.6–3.0), and pulmonologists (OR 2.1, 95% CI 1.9–2.3). Increased odds were observed for emergency room visits (OR 1.2), hospital visits (OR 1.2), and use of other community services.

### 3.3. Medication Use

Medication use in pediatric patients with AD compared to the matched control group during 2024 is detailed in Table 3, including topical treatments, phototherapy, and systemic treatments. Among children with AD, 144 (0.11%) were treated with immunosuppressive drugs as compared to 78 (0.06%) of the control group (OR 1.8, 95% CI 1.4–2.4, *p*-value < 0.001). Biologic treatments were used in 410 children with AD (0.32%) as compared to 12 (0.01%) of the control group (OR 34.3, 95% CI 19.3–60.9, *p*-value < 0.001). JAK inhibitors were used in 34 children with AD (0.03%) and 2 children (0.002%) in the control group (OR 17, 95% CI 4.1–70.8, *p*-value < 0.001). Systemic corticosteroids were used in 11,062 children with AD (8.6%) compared to 8300 (6.5%) in the control group (OR 1.4, 95% CI 1.3–1.4, *p*-value < 0.001). The use of systemic medications stratified by age distribution appears in Table 4. Medication use in pediatric patients with AD according to disease severity is detailed in Table 5.

## 4. Discussion

In this large population-based study (128,239 pediatric patients), we described healthcare utilization and medication use in children with AD. Our findings highlight the substantial healthcare burden in pediatric AD patients, as evidenced by the increased utilization of healthcare services. Children with AD demonstrated increased rates of visits to pediatricians, general practitioners (GPs), and dermatologists as compared to the control group, demonstrating the considerable medical care needs of this population.

Topical and systemic treatment use in pediatric patients with AD was increased as compared to the matched control group, including TCS, TCI, PDE4 inhibitors, phototherapy, systemic corticosteroids, immune-suppressive agents, JAK inhibitors, and biological treatments.

Previous studies demonstrated increased health care utilization and medication use in patients with AD, correlating with disease severity [12,13,14,15]. However, these studies focused on an adult AD population, with limited focus on pediatric cohorts. In comparison, a Danish population-based study, with a similar cohort as this study, reported a two-fold higher use of systemic immunosuppressives, including methotrexate, azathioprine, and cyclosporine, in children with AD [16]. This difference suggests under-utilization of systemic treatments among children with AD in Israel. It is possible that systemic therapies are used more frequently in Denmark. Both countries have public health systems: in Israel, healthcare is provided by four competing healthcare providers, with CHS being the largest healthcare provider. While treatment in both countries is provided by community clinics and hospital dermatology departments, Denmark makes higher use of community-referred hospitals [17]. In Israel, a substantial proportion are managed in community clinics. These structural differences in access to healthcare services, treatment availability, and prescribing practices may account for the variations in treatment patterns observed between the two countries.

The increased use of dupilumab in clinical practice corresponds with its recent approval as a first-line systemic treatment for severe AD (i.e., Investigator’s Global Assessment, score 4) in children aged 6 months to 12 years of age, whose disease is inadequately controlled with topical therapies. Compared to conventional immunosuppressive therapies, dupilumab offers a more favorable safety profile which may be suitable for long-term treatment [18]. A cohort study from the Netherlands, which included 502 children with atopic dermatitis, assessed drug survival of systemic drugs in children with atopic dermatitis [19]. In this study, it was observed that dupilumab had superior survival, as compared to methotrexate and cyclosporine. These findings are further supported by real-world data from the PEDISTAD registry, which demonstrated a significantly lower four-year discontinuation rate with dupilumab (14.9%) as compared to methotrexate (45.7%) and cyclosporine (63.7%) in children under 12 years of age [20]. Additionally, laboratory monitoring is not required during dupilumab treatment, as opposed to immunosuppressive treatments, contributing to treatment adherence.

The use of JAK inhibitors was low in our study, which may be attributed to the limited indication for these agents in the pediatric population in Israel. According to the criteria set by the Ministry of Health, upadacitinib is included in the health basket for adolescents aged 12 years and older and is indicated only as a second-line treatment following the failure of conventional systemic treatments, such as methotrexate, after a minimum of three months of treatment. Moreover, as for 2024, upadacitinib was the only JAK inhibitor available for patients under the age of 18 years in Israel. We expect the use of JAK inhibitors will increase in clinical practice, as recent clinical trials have demonstrated their efficacy and well-tolerated safety profile in adolescents with moderate-to-severe AD, supporting their potential for long-term use in this population [21,22,23].

Some of the ORs observed in our analysis, particularly for advanced topical and systemic treatments (e.g., TCI, PDE4 inhibitors, and biologic drugs) were markedly elevated. In CHS, the use of these agents requires prior authorization. Consequently, patients in the control group, without AD, almost did not receive these drugs, which could explain the high OR values observed.

The strengths of the study include the use of the CHS database, which enabled access to a large study population with access to data in primary care and community settings. The CHS database reports accurate real-life observations about healthcare utilization and drug use in the pediatric population with AD.

The limitations of our study include lack of severity assessment by clinical scoring systems, such as the SCORAD or IGA index, which are widely used in clinical trial settings. However, routine clinical practice in CHS does not use standardized severity measurements. To address this limitation, we utilized dispensed AD-related pharmacologic medications as a surrogate measure for disease severity. This method may lead to misclassification, as the severity of AD may not always correlate with the medication prescribed.

The diagnosis of AD in our study is based on a clinical assessment by dermatologists. The use of over-the-counter drugs or medicines purchased at private pharmacies is not included in the study, since they are not recorded in CHS database.

Despite these limitations, our study provides important data about the burden of healthcare service utilization and the drug use of patients with AD in a general healthcare setting.

In conclusion, we observed increased use of healthcare services and medications in children with AD as compared to a control group. The use of immunosuppressive agents and advanced therapies such as biologic agents and JAK inhibitors was low, as compared to a similar study in Denmark. Our findings demonstrate the substantial burden of AD in a pediatric population and suggest that the use of systemic medications should be considered in patients with moderate-to-severe disease.

## Figures and Tables

**Table 1:** Baseline characteristics of the study population.

	AD (n = 128,239) n (%)	Controls (n = 128,239) n (%)
Age, years, mean (SD)	9.7 (4.9)	9.7 (4.9)
0–4	23,073 (18.0)	23,073 (18.0)
5–9	38,940 (30.4)	38,940 (30.4)
10–14	38,562 (30.1)	38,562 (30.1)
15–18	27,664 (21.6)	27,664 (21.6)
Sex		
Females	60,835 (47.4)	60,835 (47.4)
BMI, mean (SD)	21.6 (73.1)	21.7 (81.3)
Ethnicity		
Arabs	27,494 (21.4)	27,494 (21.4)
Orthodox Jewish	9687 (7.6)	9687 (7.6)
Jewish	90,691 (70.7)	90,691 (70.7)
Other	367 (0.3)	367 (0.3)
Socioeconomic status		
Low	46,695 (36.8)	46,695 (36.8)
Medium	65,682 (51.8)	65,682 (51.8)
High	14,518 (11.4)	14,518 (11.4)
Severity AD		
Mild	114,676 (89.4)	NA
Moderate	13,019 (10)	NA
Severe	544 (0.42)	NA

Abbreviations: AD, atopic dermatitis; SD, standard deviation; NA, not applicable; n, number.

**Table 2:** Healthcare service utilization in children with atopic dermatitis and control group during 2024.

	AD (n = 128,239)	Controls (n = 128,239)	AD (n = 128,239)	Controls (n = 128,239)	
	Mean Visits (SD)	Mean Visits (SD)	n (%)	n (%)	OR (CI 95%)
Community services					
GPC clinic visits	1.8 (3.3)	1.5 (2.9)	61,787 (48.2)	56,293 (43.9)	1.2 (1.2–1.2)
Pediatric clinic visits	4.8 (5.8)	3.8 (5.0)	96,659 (75.4)	89,824 (70.0)	1.3 (1.3–1.3)
Internal medicine clinic visits	0.03 (0.25)	0.03 (0.23)	33,927 (26.5)	27,764 (21.7)	1.3 (1.3–1.3)
Dermatologist clinic visits	0.5 (0.9)	0.2 (0.6)	3346 (2.6)	2730 (2.1)	1.2 (1.2–1.3)
Allergist clinic visits	0.05 (0.43)	0.02 (0.25)	41,564 (32.4)	18,545 (14.5)	2.8 (2.8–2.9)
Otolaryngologist clinic visits	0.21 (0.63)	0.15 (0.53)	23,852 (18.6)	19,209 (15.0)	1.3 (1.3–1.3)
Pulmonologist clinic visits	0.02 (0.19)	0.01 (0.13)	4176 (3.3)	1528 (1.2)	2.8) 2.6–3.0)
Ophthalmologist visits clinic visits	0.23 (0.57)	0.18 (0.49)	18,088 (14.1)	13,787 (10.8)	1.4 (1.3–1.4)
In- and out-patient services					
Number of hospitalization days	0.13 (2.29)	0.13 (2.43)	NA	NA	NA
Emergency room visits	0.20 (0.54)	0.17 (0.49)	19,441 (15.2)	16,815 (13.1)	1.2 (1.2–1.2)
Number of hospital visits	0.037 (0.265)	0.033 (0.267)	3812 (3.0)	3309 (2.6)	1.2 (1.1–1.2)

Abbreviations: AD, atopic dermatitis; GPC, general practitioner; n, number; SD, standard deviation; CI, confidence interval; OR, odds ratio; NA, not applicable.

**Table 3:** Medication use in children with atopic dermatitis and control group during 2024.

	AD (n = 128,239) n (%)	Controls (n = 128,239) n (%)	OR (CI 95%)	*p*-Value
Topical treatments				
Topical calcineurin inhibitors	3271 (2.6)	164 (0.1)	20.4 (17.5–23.9)	<0.001
Topical PDE4 inhibitors	1303 (1.02)	50 (0.04)	26.3 (19.8–34.9)	<0.001
Topical corticosteroids	32,726 (25.5)	12,138 (9.5)	3.3 (3.2–3.4)	<0.001
Phototherapy	222 (0.17)	31 (0.02)	7.2 (4.9–10.4)	<0.001
Systemic treatments				
Antihistamines	27,670 (21.6)	15,726 (12.3)	2.0 (1.9–2.0)	<0.001
Immunosuppressive drugs	144 (0.11)	78 (0.06)	1.8 (1.4–2.4)	<0.001
Biologic drugs	410 (0.32)	12 (0.01)	34.3 (19.3–60.9)	<0.001
JAK inhibitors	34 (0.03)	2 (0.002)	17.0 (4.1–70.8)	<0.001
Systemic corticosteroids	11,062 (8.6)	8300 (6.5)	1.4 (1.3–1.4)	<0.001

Abbreviations: AD, atopic dermatitis; n, number; OR, odds ratio; PDE4, phosphodiesterase-4; JAK, Janus-kinase.

**Table 4:** Systemic medications in children with atopic dermatitis and controls during 2024, stratified by age.

Age	0–4 Years n (%)		5–9 Years n (%)		10–14 Years n (%)		15–18 Years n (%)	
	AD	Controls	AD	Controls	AD	Controls	AD	Controls
Immunosuppressive drugs	15 (0.07)	8 (0.03)	36 (0.09)	15 (0.04)	49 (0.13)	29 (0.08)	44 (0.2)	26 (0.1)
Biologic drugs ^a^	79 (0.34)	2 (0.01)	135 (0.35)	6 (0.02)	111(0.3)	2 (0.01)	85 (0.3)	2 (0.01)
JAK inhibitors	1 (0.004)	0	1 (0.003)	0	18 (0.05)	0 (0.0)	14 (0.1)	2 (0.01)

Abbreviations: ^a^, dupilumab; AD, atopic dermatitis; n, number; JAK, Janus-kinase.

**Table 5:** Medication use in children with atopic dermatitis during 2024, according to disease severity.

	Mild AD (n = 114,676)	Moderate AD (n = 13,019)	Severe AD (n = 544)
Topical treatments			
Topical corticosteroids	20,298 (17.7)	12,053 (92.6)	375 (68.9)
Topical calcineurin inhibitors	0	3082 (23.7)	189 (34.7)
Topical PDE4 inhibitors	0	1248 (9.6)	55 (10.1)
Phototherapy	0	196 (1.5)	26 (4.8)
Systemic treatments			
Systemic corticosteroids	8623 (7.5)	2315 (17.8)	124 (22.8)
Immunosuppressive drugs	0	0	144 (26.5)
Biologic drugs ^a^	0	0	410 (75.4)
JAK inhibitors	0	0	34 (6.3)

Abbreviations: ^a^, dupilumab; AD, atopic dermatitis; n, number; PDE4, phosphodiesterase-4; JAK, Janus-kinase.

## Data Availability

The data presented in this study are not publicly available due to privacy restrictions.

## Supplementary Materials

The following supporting information can be downloaded at https://www.mdpi.com/article/10.3390/jcm14186402/s1. Table S1: Residential distribution of patients with AD and controls.
