# Supplementary material for: Healthcare Service Utilization and Medication Use in 128,239 Children with Atopic Dermatitis in Israel—A Cross-Sectional Case-Control Study
Source: J Clin Med. 2025 Sep 10;14(18):6402. doi: 10.3390/jcm14186402 (PMC12470608; doi:10.3390/jcm14186402)
Supplement: Supplementary file 1 [file jcm-14-06402-s001.zip › jcm-3799409-supplementary.pdf]

Supplementary Table S1. Residential distribution of patients with AD and controls.

	AD n (%)	Controls n (%)
**Ashkelon**	7,379 (5.75)	7,417 (5.78)
**Be'er Sheva**	10,216 (7.97)	10,179 (7.94)
**Golan**	429 (0.33)	424 (0.33)
**Hasharon**	7,048 (5.50)	7,063 (5.51)
**Hadera**	8,598 (6.70)	8,540 (6.66)
**Haifa**	6,987 (5.45)	6,995 (5.45)
**Yehuda**	5,693 (4.44)	5,838 (4.55)
**Yizrael**	8,932 (6.97)	8,961 (6.99)
**Jerusalem**	12,011 (9.37)	11,834 (9.23)
**Kinneret**	1,268 (0.99)	1,325 (1.03)
**Western Negev South**	4,673 (3.64)	4,656 (3.63)
**Western Negev North**	372 (0.29)	341 (0.27)
**Akko**	9,507 (7.41)	9,483 (7.39)
**Petah Tikva**	14,208 (11.08)	14,100 (11.00)
**Safed**	1,222 (0.95)	1,263 (0.98)
**Rehovot**	9,685 (7.55)	9,552 (7.45)
**Ramla**	6,406 (5.00)	6,477 (5.05)
**Tel Aviv**	13,291 (10.36)	13,285 (10.36)
**Missing data**	314 (0.24)	506 (0.39)
**Total sum**	128,239	128,239
